# Sensory information in children’s statements of sexual abuse

**DOI:** 10.1080/20961790.2020.1814000

**Published:** 2020-09-29

**Authors:** Gérard Niveau

**Affiliations:** Geneva University Hospitals, Geneva, Switzerland

**Keywords:** Forensic sciences, forensic psychiatry, forensic psychology, credibility, sensory information, children’s statements, sexual abuse

## Abstract

The credibility of children’s statements of sexual abuse is a controversial issue in forensic psychiatry and psychology. Neurobiological and clinical laboratory studies show that real memories contain more information regarding sensory details than false memories. The goal of the present field study was to evaluate whether sensory information was present in children’s statements of sexual abuse, and whether this information was more often present in credible statements compared with non-credible statements. Sensory details were extracted from a sample of 96 statements of sexual abuse from children; 62 statements were considered credible and 34 statements were considered non-credible. This study showed that sensory information was present in 79% of children’s reports of child sexual abuse. Sensory information was significantly more often present in statements considered credible compared with non-credible statements (85.5%, *P* < 0.001), but there were large variations in the sense involved. Logistic regression analysis showed that the presence of at least one sensory detail may be a good predictor of credibility (odds ratio, OR = 23.484, *P* < 0.05). It seems appropriate to include sensory details when assessing the credibility of children’s statements of child sexual abuse, but it has not yet been demonstrated that use of such details significantly improves the validity of credibility assessments.

## Introduction

Statements of sexual abuse made by children have always posed a problem of legal validity [1]. Since the middle of the 20th century, different methods have been devised to allow more objectivity in the assessment of these statements [2]. Several kinds of credibility criteria have been proposed by different research teams at different times and in different countries. Currently, it is recognised that the most objective criteria are those concerning analysis of the statement content [3].

A commonly used and validated credibility assessment method is Criteria-Based Content Analysis (CBCA) [4]. This method was conceived by Undeutsch in the 1950s and structured in its current version by Steller and Koehnken (1989). CBCA forms the main component of the Statement Validity Assessment (SVA) method, which comprises three stages: verification of the quality of the child’s interview, appraisal of the CBCA items and evaluation of the outcome with the Validity Checklist [2]. CBCA items (Table 1) cover the general characteristics of the interview (items 1–3), specific content of the statement (items 4–13), content related to the motivation for the statement (items 14–18) and specific elements of the crime (item 19). CBCA laboratory and field studies reported a validity of approximately 75% [5] and an inter-rater reliability of approximately 80% [6]. Some studies showed that implementation of the Validity Checklist could slightly improve the validity of CBCA results [7], but this did not exceed 80% [2]. Increasing the validity of this tool would have important implications for legal proceedings as well as for child protection and therapeutic interventions [8].

**Table 1.** The 19 criteria of the Criteria-Based Content Analysis (CBCA) scale and their frequencies.

Criterion number	Denomination	Presence in the 96 cases (*n*, %)
1	Logical structure	66 (68.8)
2	Unstructured production	77 (80.0)
3	Quantity of details	73 (76.0)
4	Contextual embedding	80 (83.3)
5	Descriptions of interactions	85 (88.5)
6	Reproduction conversations	52 (54.2)
7	Unexpected complications	28 (29.2)
8	Unusual details	20 (20.8)
9	Superfluous details	26 (27.1)
10	Misunderstood accurate details	16 (16.7)
11	Related external associations	21 (21.9)
12	Subjective mental state	40 (41.7)
13	Perpetrator’s mental state	26 (27.1)
14	Spontaneous corrections	30 (31.3)
15	Admitting lack of memory	26 (27.1)
16	Doubting own testimony	16 (16.7)
17	Self-deprecation	14 (14.6)
18	Pardoning the perpetrator	8 (8.3)
19	Characteristic details	49 (51.0)

The CBCA criteria were designed on the basis of clinical observations, but were not based on a theoretical concept [9]. However, research on memory has shown that the memory of actual facts differs from the memory of imaginary facts [10]. According to the theory of reality monitoring, memories of facts actually experienced contain sensory details, unlike memories of imaginary facts, which are characterised by mental processes [11]. Functional magnetic resonance imaging research has confirmed this construct [12, 13]. Based on this theory, the Reality Monitoring method was designed to distinguish between true and false memories. This method evaluates eight items: clarity, perceptual information, spatial information, temporal information, affect, reconstructability of the story, realism and cognitive operations [2]. Most items in this method are the same as those in the CBCA. However, the item “perceptual information” is not included among the CBCA items.

The Reality Monitoring method has not been scientifically validated and therefore cannot be used in a forensic context. The CBCA scale remains the only method usable in a forensic context, but this scale requires improvement to increase its validity [5]. However, it may be possible to improve the CBCA by including the sensory information criterion from the Reality Monitoring method.

The main goal of this study was to establish how often sensory information was present in children’s sexual abuse statements in a field context. This study also aimed to determine whether sensory information was more frequent in credible statements compared with non-credible statements, and whether there were correlations between this information and other factors such as the child’s age or the nature of the facts described.

## Material and methods

### Sample

An initial sample of 127 files was created that included all forensic records concerning the credibility assessment of sexual abuse cases reported by children between 1 January 2015 and 31 December 2018 in Geneva, Switzerland. The files were obtained from the University Centre of Legal Medicine in Geneva. Each file contained criminological information about the facts of the case, sociodemographic and medical information about the alleged abuse victim, the child’s statement verbatim and the credibility assessment of the statement. All statements were the result of first time, single session interviews by specialised police. All interviews were conducted using the standardised National Institute of Child Health and Human Development (NICHD) protocol [14].

Records were excluded if the child’s statement was technically unusable, the child did not report sexual abuse, the child had a serious mental illness or the credibility decision was absent. Regarding this last point, to reduce the risk that the credibility assessment was inaccurate, cases in which the credibility decision was not confirmed by at least one piece of evidence other than the credibility assessment itself were excluded. Evidence that could confirm credibility or non-credibility included a confession, retraction, material element, medical element or confirmed testimony.

After these exclusions, the sample included in the analysis comprised 96 files. The children were aged between 6 and 16 years, with a mean age of 11 years and a median age of 10 years. There were 46 males and 50 females. Distinctions were made between acts with penetration versus acts without penetration and single acts versus multiple acts to clarify relationships between the type and number of alleged acts and the presence of sensory details. Greater precision was not possible given the content of the files.

There were 42 alleged cases (43.8%) of acts without sexual penetration and 54 alleged cases (56.2%) of acts with sexual (vaginal, anal or oral) penetration. The alleged abuses consisted of a single act for 46 cases (47.9%) and more than one act for 50 cases (52.1%). Of the 96 total cases, 62 cases (64.6%) were considered credible and 34 cases (35.4%) were non-credible. The distribution of CBCA criteria identified by the experts is detailed in Table 1.

### Procedure and variables

In each file, the child’s age and gender, type of abuse, number of abuse events and credibility assessment of the statement were identified as independent variables. The credibility assessment reports had been completed before the study by two experts working together. These experts were a psychologist and a psychiatrist trained in using the SVA method and that had been completing these assessments for more than 5 years. The experts were independent of the judiciary and the police. For each case, the two experts first assessed the credibility of the statement independently (percentage of agreement, 78%). Next, they coordinated their results to give a final result.

Independent credibility variables were identified in the files. The rating of the CBCA criteria and the final credibility decision were noted for each case. The dependent data were the sensory details in the children’s verbatim statements. For this research, the investigator read each statement verbatim twice and recorded the sensory details noted in each statement. The investigator did not know whether the statement was considered credible or not credible by the experts.

All details referring to direct perception of one of the five senses were included. Reality monitoring theory suggests “memories about imagined events are derived from an internal source and are therefore likely to contain cognitive operations, such as thoughts and reasonings” [2]. Details that resulted from a mental elaboration were therefore excluded. The sensory details for each sense were defined as follows.

Smell details: The child mentioned an odour during the period of abuse. Statements such as “it stank”, “he did not smell good” and “I remember his cologne” were retained. Expressions such as “there was a bad mood” or “the air was not nice” were not retained.

Taste details: The child mentioned a taste during the period of abuse. Statements such as “it was disgusting” and “it made me want to throw up” were retained. Expressions such as “it was in my mouth” and “I could not swallow anymore” were not retained.

Sound details: Two types of sound details were selected. Details of conversations reported, such as “he said ‘walk in front of me'” or “he sang ‘In the Moonlight'”, were retained. In addition, sound details that were not reminders of conversations, such as “he spoke loudly”, “there was music” or “I heard the song of birds”, were retained. However, expressions such as “he spoke only of himself” or “he told me with malice” were not retained.

Physical sensation details: This information concerned touch and internal sensations during the period of abuse. Statements such as “it was sweet”, “it was rough”, “it hurt me”, “I choked” and “it made me hiccup” were retained. Expressions such as “quick contact”, “I was tired” and “he put his hand on me” were not retained.

Visual details: The child reported seeing something during the period of abuse. Details such as “he made a grimace”, “he had a very red face” and “the light was bright” were retained. Expressions such as “he came into the room”, “the house was big” and “there were two of them” were not retained.

### Statistical analysis

The statements were first classified as having at least one sensory detail or not having such details, and then as credible or not credible. Descriptive statistics and between-group comparisons for categorical variables were analysed using chi-square tests. Because of the structure of the sample and to allow two subgroups of the same size, the median age of 10 years was used to distinguish younger children and older children. Next, binary logistic regression analysis was conducted using the significant variables as predictors of credible statements. The two-tailed level of statistical significance was set at 0.05. All analyses were performed using SPSS version 25 (IBM Corp., Armonk, NY, USA).

## Results

### Bivariate relationships between variables and sensory details

Of the 96 cases, sensory details were found in 76 statements (79%). Table 2 shows the distribution of sensory details according to age, gender, type of abuse, number of abuse events and the expert-assessed credibility. Sensory details were more frequent in the statements of children aged over 10 years, statements of female children and when the statements reported acts of sexual penetration, but these differences were not significant. Sensory details were significantly more frequent in statements concerning a declaration of only one sexual abuse event, which was not an expected outcome. Sensory details were significantly more often present in statements that had been considered credible by the experts.

**Table 2.** Descriptive statistics of the present sample based on the presence of sensory details in the statements (*N*=96).

	Age (*n*, %)		Gender (*n*, %)		Type of abuse (*n*, %)		Number of abuse events^a^ (*n*, %)		Credibility^a^ (*n*, %)	
	<10	≥10	Female	Male	No penetration	Penetration	One	More than one	Credible	Not credible
At least one sensory detail (*n=*66)	30 (45.5)	36 (54.5)	38 (57.6)	28 (42.4)	25 (37.9)	41 (62.1)	38 (57.6)	28 (42.4)	53 (80.3)	13 (19.7)
No sensory details (*n=*30)	18 (60.0)	12 (40.0)	12 (40.0)	18 (60.0)	17 (56.7)	13 (43.3)	8 (26.7)	22 (73.3)	9 (30.0)	21 (70.0)

^a^*P*<0.05.

### Bivariate relationships between sensory details and credibility

The systematic survey of the sensory details present in the statements showed variability according to the sense involved. Table 3 shows the distribution of sensory details according to the expert credibility assessment. Taste and smell details were rare and almost exclusively present in statements deemed credible. Sound details were frequent but mainly consisted of conversation reminders. Visual details and physical sensations were significantly more common in statements considered credible than in statements considered non-credible. Because of the small numbers of cases in some subgroups, statistical significance was not investigated for each type of sensory detail.

**Table 3.** Presence of sensory details in statements considered credible and non-credible according to the statement validity assessment method.

	At least one taste detail	At least one smell detail	At least one auditory detail	At least one auditory detail not including conversations	At least one visual detail	At least one physical sensation detail	At least one sensory detail
Credible statements (*n=*62)	2 (3.2%)	4 (6.5%)	45 (72.6%)	17 (27.4%)	10 (16.1%)	19 (30.6%)	53 (85.5%)
Non-credible statements (*n=*34)	0	1 (2.9%)	13 (38.2%)	4 (11.8%)	3 (8.8%)	3 (8.8%)	13 (38.2%)

### Bivariate relationships between CBCA items and credibility

Table 4 shows the presence of the 19 CBCA criteria and the additional sensory details criterion in the statements considered credible and non-credible by the experts. All criteria were more frequently present in the statements considered credible than in those considered non-credible. Criteria 1 (logical structure), 3 (quantity of details), 4 (contextual embedding), 6 (reproduction conversations), 12 (subjective mental state) and 19 (characteristic details) of the CBCA list and the additional criterion of “at least one sensory detail” were significantly more frequent in statements considered credible than in non-credible statements.

**Table 4.** Presence of Criteria-Based Content Analysis criteria and sensory details in credible and non-credible statements.

Present criteria	Credible statements (*n* = 62)	Non-credible Statements (*n* = 34)	*P*-value
CBCA 1	51 (82.3%)	15 (44.1%)	<0.001
CBCA 2	53 (85.5%)	24 (70.6%)	NS
CBCA 3	55 (88.7%)	18 (52.9%)	<0.001
CBCA 4	57 (91.9%)	23 (67.6%)	<0.01
CBCA 5	60 (96.8%)	25 (73.5%)	NS
CBCA 6	40 (64.5%)	12 (35.5%)	<0.01
CBCA 7	20 (32.3%)	8 (23.5%)	NS
CBCA 8	15 (24.2%)	5 (14.7%)	NS
CBCA 9	18 (29.0%)	8 (23.5%)	NS
CBCA 10	12 (19.4%)	4 (11.8%)	NS
CBCA 11	16 (25.8%)	5 (14.7%)	NS
CBCA 12	32 (51.6%)	8 (23.5%)	<0.01
CBCA 13	18 (29.0%)	8 (23.5%)	NS
CBCA 14	22 (35.3%)	8 (23.5%)	NS
CBCA 15	17 (27.4%)	9 (26.5%)	NS
CBCA 16	11 (17.7%)	5 (14.7%)	NS
CBCA 17	12 (19.4%)	2 (5.9%)	NS
CBCA 18	6 (9.7%)	2 (5.9%)	NS
CBCA 19	42 (67.7%)	7 (20.6%)	<0.001
At least one sensory detail	53 (85.5%)	13 (38.2%)	<0.001

NS: no statistical significance.

### Model for predicting credibility

Given the results obtained in bivariate analyses, the number of abuse events, the presence of at least one sensory detail and CBCA criteria 1, 3, 4, 6 and 12 appeared to be important variables to be included in the binary regression analysis. Criterion 19 of the CBCA was excluded because it was an unquantifiable subjective criterion. The results of the logistic regression analysis for identifying predictors of credibility are shown in Table 5. The model was significant (*P* < 0.001) and correctly classified in 84.4% of the cases, with Nagelkerke’s *R*^2^=0.618. The analysis confirmed that the number of abuse events was a low-power negative indicator of credibility. The most powerful predictor of credibility was CBCA criterion 4, and the presence of at least one sensory detail appeared to be a good predictor (odds ratio, OR = 23.484, *P* < 0.05).

**Table 5.** Binary logistic regression analysis predicting credibility.

Predictors	B	SE	OR	95%CI	*P-*value
More than one abuse event (yes *vs.* no)	−0.356	0.697	0.701	0.179–2.746	0.610
CBCA 1 (present *vs.* absent)	1.899	0.722	6.680	1.622–27.505	0.009
CBCA 3 (present *vs.* absent)	2.211	0.743	9.123	2.126–39.142	0.003
CBCA 4 (present *vs.* absent)	2.492	0.809	12.083	2.474–59.012	0.002
CBCA 6 (present *vs.* absent)	1.009	1.527	0.364	0.018–7.261	0.508
CBCA 12 (present *vs.* absent)	0.394	0.660	1.483	0.407–5.403	0.550
At least one sensory detail (yes *vs.* no)	3.156	1.577	23.484	1.069–516.130	0.045

B: regression coefficient; SE: standard error; OR: odds ratio; CI: confidence interval; CBCA: Criteria-Based Content Analysis.

## Discussion

The main result of this study was that in a sample of sexual abuse statements collected in a field context, sensory information was significantly more frequent in credible statements than in non-credible statements. This result confirmed past and recent laboratory studies about children’s and adult’s testimonies [10, 15–17]. The presence of one or more sensory details in a child’s statement may be considered a factor of credibility; however, it is not a characteristic factor. The value of the sensory details was of the same order as the other factors found in the CBCA or Reality Monitoring lists. They must be taken into consideration in quantitative and qualitative aspects.

Regarding the quantitative aspect, the number of sensory details present in a statement had a similar value to the number of details of another kind. This number must be assessed according to the characteristics of the event described and according to the cognitive abilities of the child. For example, many details cannot be expected for a brief, one-time event [9]. Similarly, fewer details are expected for a very young child (e.g. aged 6 years) than for an older child (e.g. aged 15 years) [17]. The number of sensory details expected to increase credibility must therefore be assessed according to the specific contextual factors of the statement.

Regarding the qualitative aspect, this study clearly showed that the kind of sensory details mentioned by children in police interviews were not present in the same way in relation to each other. Inclusion of smell and taste details were rare, whereas information about sounds was common. Similar findings have previously been described in general memory studies [18]. However, these differences are of particular importance in the medico-legal framework. Indeed, in the credible statements, details of taste and smell were rare, but these details were almost always absent in non-credible statements. This finding makes the statistical analysis of the role of these details in the assessment of credibility impossible. However, for CBCA scale designers, some rare details, such as “unusual details” or “pardoning perpetrator”, are very supportive of credibility when they are present in a statement [19]. If rare sensory details can be considered in the same way as the rare criteria of the CBCA scale, we can presume that if a smell or taste detail is present in a child abuse statement, this detail has strong importance in favour of credibility. Sound details were also significantly more frequent in the credible statements. However, if conversation reminders were excluded from sound details, sound details were almost completely absent from non-credible statements and weakly present in the credible statements (similar to smell and taste details). Using the same reasoning as for smell and taste details, we can therefore consider that sound details (except conversation reminders) are also important in favour of credibility.

This study showed that visual and physical sensation details were sensory details whose presence in a statement were the most clearly indicative of credibility. This result confirmed previous laboratory studies, particularly those based on biological knowledge of memory [12, 20]. These findings are important because the activation of visual and physical perceptions is often present in actual sexual abuse cases. These details will therefore often be cited by an actual victim, whereas they will often not be mentioned if there has not been real interaction [21]. It is therefore valid to include the presence of one or more visual details or physical sensations to the list of factors favourable to the credibility of child sexual abuse statements.

A limitation of this study was that the assessment of credibility by experts using the CBCA scale is not completely reliable. With the method used in the cases included in this study, the assessment of credibility had a validity of approximately 75%–80% compared with reality [2], which might have affected the degree of validity of the sensory criteria for credibility. To limit this confounding factor, this study selected statements whose credibility or non-credibility was confirmed by external and objective factors. Therefore, the two groups of statements (credible and non-credible) can be considered more than 80% valid. However, this validity does not reach 100%.

Another limitation was the small size of some subgroups. For example, there were insufficient occurrences of taste or smell details in credible and non-credible statements to allow a statistical approach. For this reason, qualitative estimates rather than quantitative conclusions were drawn in these subgroups. A limitation can also be mentioned with regard to the collection of sensory information by a single investigator. However, this data collection constituted a direct rather than a subjective observation. Use of two assessors was not justified because the risk that the evaluator forgot to address some sensory information after two successive readings was low.

In conclusion, this study shows that the sensory details in children’s statements can be considered an important factor related to credibility. However, for methodological and sample size reasons, it was not possible to expand the research to determine whether the inclusion of a sensory detail criterion in the CBCA list would significantly alter the results of the credibility versus assessment not using this criterion. At this stage, the findings of this study cannot be considered applicable to the decision-making for a single case. The lack of sensory details in any given child statement does not inherently mean the statement is not credible. The ultimate goal of this research on sensory details in children’s statements of sexual abuse was to improve the validity of credibility assessment methods, particularly the SVA method. Further studies should therefore compare the results using the CBCA scale with and without scores for sensory details by two independent research groups.
